# Gender diversity, corporate governance and firm-specific data of all public listed US firms during 2000–2019

**DOI:** 10.1016/j.dib.2024.110328

**Published:** 2024-03-20

**Authors:** Nafisah Yami, Hashem Alshurafat

**Affiliations:** a Accounting Department, College of Business Administration, King Saud University, Riyadh 12372, Saudi Arabia; b Department of Accounting, Business School, The Hashemite University, Zarqa, Jordan

**Keywords:** Financial performance, Corporate governance, Female leadership, United States

## Abstract

This article provides in-depth data on gender diversity, corporate governance practices and specific firm factors. The study compiles panel data for all publicly listed companies in the United States, featured in the S&P index from 2000 to 2018, except for financial and utilities firms. The data set includes variables regarding gender diversity, board characteristics, firm performance, and other crucial factors. The data was extracted from the annual reports of each firm using Compustat and BoardEX databases. Researchers can apply the data in assessing the impact of appointing female directors on the value of a firm through event studies. Moreover, the dataset can help address endogeneity issues, enabling researchers to control for potential confounding variables and draw more accurate conclusions.

Specifications Table

**Table:** 

Subject	Finance
Specific subject area	Corporate finance, Corporate governance.
Data format	Raw, Filtered, Analyzed.
Type of data	Excel file
Data collection	Data is obtained from the BoardEX database and Compustat database.
Data source location	All publicly listed US firms over 19 years from 2000 to 2018 in the S&P index.
Data accessibility	Repository name: Mendeley Data Data identification number: 10.17632/fdw347mttz.1 Direct URL to data: https://data.mendeley.com/datasets/fdw347mttz/1
Related research article	Yami, N., Poletti-Hughes, J. and Hussainey, K. (2023), “The impact of female directors on earnings management and the moderating effect of board quality: enabler or deterrent?”, Journal of Financial Reporting and Accounting, In Press. https://doi.org/10.1108/JFRA-03-2023-0119.

## 1 Value of the Data

• This dataset provides researchers with the opportunity to analyze the correlation between the presence of female directors and a company's financial performance.
• The dataset offers a comprehensive view of gender diversity in the corporate world, enabling a better understanding of the global status of female directors and trends in their appointment to boards over time.
• By providing variables related to board characteristics, firm performance, and other relevant factors, the dataset can assist in addressing endogeneity issues, allowing researchers to control for potential confounding variables and draw more accurate conclusions.
• The dataset can also be utilized to evaluate the signalling effects of the appointment of female directors on firm value through event studies.

## 2 Data Description

The dataset provides insights into corporate governance and finance. It enables researchers to examine gender diversity in boardrooms, firm performance and characteristics, board composition and independence, and instrumental variables. These factors can help researchers uncover patterns, relationships, and implications for corporate practices and performance [4,6].

This dataset covers all publically listed companies in the United States from 2000 to 2018 which are listed in the S&P index. The starting point of 2000 is due to the minimal data available in the BoardEX database before this time in relation to board directors’ information. Compustat is the source of financial data. As previous research indicates, financial and utilities firms are excluded from the sample due to their distinct regulations, which expose their directors to liability risks that non-financial firms are not subject to [1,7]. The sample size of non-financial firms amounts to 17,220. Financial variable outliers are adjusted to the 98 % level in accordance with Bharath and Shumway's [3] study.

Table 1 presents a comprehensive description of the variables that are provided in this data article. These variables play a crucial role in examining various aspects of corporate governance, firm performance, and the impact of gender diversity in the boardroom, making them valuable tools for empirical research in business and economics [2,5].

**Table 1:** Definition of variables.

**Firm characteristics**	

ROA	Net income before extraordinary items to total assets.
Tobin's Q	Total assets minus equity plus the market value of equity over total assets.
firm size	Logarithm of total assets.
Capital exp	Total capital expenditures scaled by total assets.
R&D	R&D expenses over the total of assets.
Cash	It is a ratio of cash and cash equivalents to total assets.
Advertisement expenses	advertising expense divided by net sales.
Leverage	Total liabilities to total assets.
Firm age	Number of years of total assets reported by Compustat since 1977.
Firm growth	Percentage growth between year t and t-1.
Distressed firm	A dummy variable when Z-Score is less than the threshold value of 1.81 as indicated by Altman) and 0 otherwise.
Dividends	A dummy variable that equals one when firms pay dividends in that given year and zero otherwise.
**Board directors’ characteristics**	
Board size	The logarithm of the total number of directors on the firm's board.
Board independence	Total number of independent directors to board size.
Directors number	Total number of directors on the board.
Independent directors	Total number of independent directors on the board.
Directors’ tenure	Average number of years in post for directors.
Directors age	Average age of directors.
CPA certificate %	Total number of directors that hold CPA over total of directors.
CPA certificate N	Total number of directors that hold CPA on the board.
Master/PhD certificate %	The total number of directors that hold Master's and PhD over the total of directors.
Master/PhD certificate N	The total number of directors that hold Master's and PhD on the board.
CEO Tenure	The average number of years that the CEO has been on the board.
CEO age	The average age that the CEO has been on the board.
CEO duality	A dummy that equals one when the CEO and Chair are the same person and zero otherwise.
All seats	Number of external seats of all directors divided by number of directors.
Male seats	Number of male external seats of male directors divided by number of male directors.
**Female directors characteristics**	
Female director %	Total number of female directors on the board divided by board size.
Female director N	Number of female directors on the board.
Independent Female director %	Number of independent female directors over board size.
Female directors age	The average age of female directors on the board.
Female master/PhD	Number of female directors with Master/PhD + certificates.
Female CPA	Number of female directors with CPA certificate.
**Instruments**	
Male connectedness to female directors	The proportion of male directors on the board who sit on other boards with at least one female director.
Female industry ratio	The proportion of female directors in the same industry based on two-digit code.
Firm visibility (S&P 500)	A dummy variable that takes one if a firm is listed in the S&P 500 and zero otherwise.

Table 2 displays descriptive statistics for the variables in the dataset, categorized into three main groups: Firm Characteristics, Board Characteristics, and Instrumental Variables. In the Firm Characteristics section, financial and operational indicators are analyzed. It is worth noting that Return on Assets (ROA) has an average of 0.09, indicating that, on average, the firms in the sample generate a 9 % return on their assets. Tobin's Q has an average of 2.05, suggesting that firms typically have a higher market value compared to their book value. Firm size is measured by the natural logarithm of total assets, with an average of 7.36. It seems that firms have moderate capital expenditure, as the capital expenditure variable has an average of 0.05. Research and development expenditure is relatively low, with an average of 0.03. In addition, the average age of firms is about 32 years, and the mean firm growth rate is 0.11.

**Table 2:** Descriptive statistics.

Variable	Obs.	Mean	Std. Dev.	Min	Max
**Firms characteristics**					
ROA	17,256.00	0.09	0.10	-0.30	0.35
Tobin's Q	17,256.00	2.05	1.26	0.71	7.65
firm size	17,256.00	7.36	1.66	3.89	11.67
Capital exp	17,256.00	0.05	0.05	0.00	0.28
R&D	17,256.00	0.03	0.06	0.00	0.29
Cash	17,256.00	0.18	0.18	0.00	0.76
Advertisement expenses	17,256.00	0.01	0.03	0.00	0.17
Leverage	17,256.00	0.48	0.20	0.08	0.92
Firm age	17,256.00	32.25	5.12	23.00	41.00
Firm growth	17,229.00	0.11	0.24	-0.45	1.20
Distressed firms	17,256.00	0.24	0.43	0.00	1.00
Dividends	17,256.00	0.51	0.50	0.00	1.00
**Board characteristics**					
Board size	17,256.00	2.15	0.25	1.61	2.71
Board independence	17,256.00	0.68	0.13	0.00	1.57
Directors number	17,256.00	8.90	2.22	5.00	15.00
Independent directors	17,229.00	6.16	2.12	2.00	12.00
Directors’ tenure	17,256.00	6.53	3.04	0.75	15.98
Directors age	14,208.00	60.98	4.42	49.29	72.14
CPA certificate %	7547.00	0.39	0.19	0.00	1.00
CPA certificate N	7547.00	3.41	1.83	0.00	11.00
Master/PhD certificate %	7547.00	0.32	0.19	0.00	1.17
Master/PhD certificate N	7547.00	2.82	1.74	0.00	12.00
CEO Tenure	17,117.00	5.68	5.69	0.10	30.00
CEO age	16,992.00	56.39	7.19	40.00	75.00
CEO duality	17,256.00	0.57	0.49	0.00	1.00
All seats	17,256.00	1.50	1.82	0.00	19.00
Male seats	17,256.00	1.29	1.59	0.00	15.00
**Female directors characteristics**					
Female director %	17,256.00	0.11	0.10	0.00	0.75
Female director N	17,256.00	1.07	1.02	0.00	4.00
Independent female ratio	17,256.00	0.09	0.09	0.00	0.54
Female directors age	4737.00	57.34	6.64	28	83
Female master/PhD	4737.00	0.59	0.67	0	4
Female CPA	4737.00	0.58	0.68	0	4
**Instruments**					
Male connectedness to female directors	17,256.00	0.98	1.56	0.00	15.00
Female industry ratio	17,256.00	0.12	0.02	0.08	0.18
Firm visibility (S&P 500)	17,256.00	0.35	0.47	0.00	1.00

The board characteristics section provides insights into the composition of corporate boards. On average, there are approximately 8.90 directors per board, with 6.16 of them being independent directors. Female directors make up around 1.08 positions, accounting for about 11 % of the total directors. The mean age of directors is around 61 years, while directors have an average tenure of 6.53 years. Board independence, measured as the ratio of independent directors, is about 0.68. The board size seems to be relatively stable, with an average of 2.15. A subset of directors holds CPA certificates and master's/PhD degrees, which may be important for board expertise. Within the instrumental variables section, the female industry ratio is around 12 %, indicating that women are not highly represented in the broader industry. Additionally, directors in your sample are also connected to external seats, with an average of 1.50 external seats.

## 3 Experimental Design, Materials and Methods

There are 12 variables related to firm characteristics, 15 variables related to board characteristics, 6 variables related to female directors’ characteristics and 3 instrumental variables. The data is categorized based on publicly listed non-financial US firms that have complete information available from the Compustat database. This information is then matched with directors’ information from BoardEx.

These variables are significant because they can be used to investigate a range of research questions in the fields of corporate governance, finance, economics, and management. Researchers can utilize these variables to study topics such as board diversity, firm performance, governance structures, and external influences. They also allow for the exploration of how factors like gender, firm size, age, and industry dynamics impact corporate behaviour and outcomes. The diversity of these variables creates a variety of research possibilities and can contribute to a deeper understanding of how different factors influence business operations and outcomes.

## Ethics Statement

The authors have read and followed the ethical requirements for publication in Data in Brief and confirmed that the current work does not involve human subjects, animal experiments, or any data collected from social media platforms.

## Funding

This research did not receive any specific grant from funding agencies in the public, commercial, or not-for-profit sectors.

## CRediT authorship contribution statement

**Nafisah Yami:** Conceptualization, Methodology, Software, Data curation, Writing – original draft. **Hashem Alshurafat:** Writing – review & editing, Visualization, Investigation, Validation, Supervision.

## Declaration of Competing Interest

The authors declare that they have no known competing financial interests or personal relationships that could have appeared to influence the work reported in this paper.

## Data Availability

Gender Diversity, Corporate Governance and Firm Specific Data of All Public Listed US Firms (Original data) (Mendeley Data).
